# Supplementary material for: High-coverage whole-genome analysis of 1220 cancers reveals hundreds of genes deregulated by rearrangement-mediated cis-regulatory alterations
Source: Nat Commun. 2020 Feb 5;11:736. doi: 10.1038/s41467-019-13885-w (PMC7002524; doi:10.1038/s41467-019-13885-w)
Supplement: Supplementary file 2 — Description of Additional Supplementary Files [file 41467_2019_13885_MOESM2_ESM.pdf]

Description of Additional Supplementary Files

File Name: Supplementary Data 1
Description: Related to Figure 1. Cancer cases examined in this study, with patientlevel annotation regarding specific molecular features.

File Name: Supplementary Data 2
Description: Related to Figure 1. Complete set of correlations between gene expression and nearby SV event, according to region examined and the regression model applied.

File Name: Supplementary Data 3
Description: Related to Figure 2. SVs associated with TERT, with associated expression and numbers of enhancer elements within a 0.5 Mb region upstream of each rearrangement breakpoint.

File Name: Supplementary Data 4
Description: Related to Figure 4. SVs associated with CD274 and PDCD1LG2, with associated expression.

File Name: Supplementary Data 5
Description: Related to Figure 5. Includes the following: 1) Numbers of enhancer elements within a 0.5 Mb region upstream of each rearrangement breakpoint, with associated enrichment patterns (for 1233 genes with at least 7 SV breakpoints 0-20kb upstream and with breakpoint mate on the distal side from the gene); and 2) Average change in distance of the nearest enhancer element in proximity to each gene, as a result of rearrangement (for 829 genes with at least 5 SV breakpoints 0-20kb upstream and with breakpoint mate on the distal side from the gene, where the breakpoint occurs between the gene start site and its nearest enhancer in the unaltered scenario).

File Name: Supplementary Data 6
Description: Related to Figure 6. Correlations between DNA methylation and gene expression, and correlations between DNA methylation and adjacent SV breakpoint event (for breakpoints occurring 0-20kb upstream).

File Name: Supplementary Data 7
Description: R source code written for this study. Code and data are provided as separate tabs within an Excel file; contents of each tab should be saved as a separate text file with the proper file extension. R code was used to generate gene-level linear models (code in “SV_0-20kb_linear model_R code” tab, using datasets in “SV_20” tab, “copy_20” tab, and “expression_20” tab, which data represents just the 384 genes significant in the SV+cancer type+copy model, with the sample and gene descriptions in the “sample_and_gene_info” tab), to carry out permutation testing of the 0-20kb upstream dataset (code in “R code_shuffle_SV_100 times” tab, using datasets in “SV_20”, “copy_20”, and “expression_20” tabs), and to generate circos plots (code in “R-code_for_circos_plots” tab, using dataset in “TERT_SVs_for_circos_plot” tab).
