# Supplementary material for: High-coverage whole-genome analysis of 1220 cancers reveals hundreds of genes deregulated by rearrangement-mediated cis-regulatory alterations
Source: Nat Commun. 2020 Feb 5;11:736. doi: 10.1038/s41467-019-13885-w (PMC7002524; doi:10.1038/s41467-019-13885-w)
Supplement: Supplementary file 10 — Reporting Summary [file 41467_2019_13885_MOESM10_ESM.pdf]

nature research

Corresponding author(s): Chad Creighton

Reporting Summary

Nature Research wishes to improve the reproducibility of the work that we publish. This form provides structure for consistency and transparency in reporting. For further information on Nature Research policies, see Authors & Referees and the Editorial Policy Checklist.

Statistical parameters

When statistical analyses are reported, confirm that the following items are present in the relevant location (e.g. figure legend, table legend, main text, or Methods section).

n/a	Confirmed	
☐	☒	The exact sample size (n) for each experimental group/condition, given as a discrete number and unit of measurement
☐	☒	An indication of whether measurements were taken from distinct samples or whether the same sample was measured repeatedly
☐	☒	The statistical test(s) used AND whether they are one- or two-sided<br>*Only common tests should be described solely by name; describe more complex techniques in the Methods section.*
☐	☒	A description of all covariates tested
☐	☒	A description of any assumptions or corrections, such as tests of normality and adjustment for multiple comparisons
☐	☒	A full description of the statistics including central tendency (e.g. means) or other basic estimates (e.g. regression coefficient) AND variation (e.g. standard deviation) or associated estimates of uncertainty (e.g. confidence intervals)
☐	☒	For null hypothesis testing, the test statistic (e.g. F, t, r) with confidence intervals, effect sizes, degrees of freedom and P value noted<br>*Give P values as exact values whenever suitable.*
☒	☐	For Bayesian analysis, information on the choice of priors and Markov chain Monte Carlo settings
☒	☐	For hierarchical and complex designs, identification of the appropriate level for tests and full reporting of outcomes
☒	☐	Estimates of effect sizes (e.g. Cohen's d, Pearson's r), indicating how they were calculated
☐	☒	Clearly defined error bars<br>*State explicitly what error bars represent (e.g. SD, SE, CI)*

Our web collection on statistics for biologists may be useful.

Software and code

Policy information about availability of computer code

Data collection	The PCAWG workflows are also available as Docker images through Dockstore enabling researchers to replicate the steps involved in the data assembly.
Data analysis	R software version 3.0 and lm function was used, with source code available as part of Supplementary Data 7.

For manuscripts utilizing custom algorithms or software that are central to the research but not yet described in published literature, software must be made available to editors/reviewers upon request. We strongly encourage code deposition in a community repository (e.g. GitHub). See the Nature Research guidelines for submitting code & software for further information.

Data

Policy information about availability of data

All manuscripts must include a data availability statement. This statement should provide the following information, where applicable:

- Accession codes, unique identifiers, or web links for publicly available datasets
- A list of figures that have associated raw data
- A description of any restrictions on data availability

All data used in this study are publicly available. The consensus SV calls are available from synapse (https://www.synapse.org/#!Synapse:syn7596712). Copy number

data are available at the ICGC Data Portal (https://dcc.icgc.org/pcawg). The gene expression dataset is available from synapse (https://www.synapse.org/#!Synapse:syn5553991).

Field-specific reporting

Please select the best fit for your research. If you are not sure, read the appropriate sections before making your selection.

☒ Life sciences ☐ Behavioural & social sciences ☐ Ecological, evolutionary & environmental sciences

For a reference copy of the document with all sections, see nature.com/authors/policies/ReportingSummary-flat.pdf

Life sciences study design

All studies must disclose on these points even when the disclosure is negative.

Sample size	In all, 2658 patients with whole genome data were represented in the PCAWG datasets, spanning a range of cancer types (bladder, sarcoma, breast, liver-biliary, cervix, leukemia, colorectal, lymphoma, prostate, eosophagus, stomach, central nervous system or “cns”, head/neck, kidney, lung, skin, ovary, pancreas, thyroid, uterus). Of the 2658 donors (Supplementary Data 1) included among the whitelist (acceptable for all analyses) and graylist (excluded from some analyses carried out as part of PCAWG-led efforts), 1220 had RNA data, 32 of which were graylisted. In accordance with the PCAWG consoritium policy, we included the graylisted cases in our analysis, as these were found to have no issues pertaining to our integration analysis approaches involving RNA and SV data.
Data exclusions	No data were excluded from the analysis.
Replication	Not applicable, no experimental data
Randomization	Not applicable, no experimental data
Blinding	Blinding not relevant, as samples were not allocated to experimental groups.

Reporting for specific materials, systems and methods

Materials & experimental systems

n/a	Involved in the study
☒	☐ Unique biological materials
☒	☐ Antibodies
☒	☐ Eukaryotic cell lines
☒	☐ Palaeontology
☒	☐ Animals and other organisms
☒	☐ Human research participants

Methods

n/a	Involved in the study
☒	☐ ChIP-seq
☒	☐ Flow cytometry
☒	☐ MRI-based neuroimaging
